# Exploring the mycobiome and arbuscular mycorrhizal fungi associated with the rizosphere of the genus *Inga* in the pristine Ecuadorian Amazon

**DOI:** 10.3389/ffunb.2023.1086194

**Published:** 2023-03-03

**Authors:** Valentina Arévalo-Granda, Aileen Hickey-Darquea, Belén Prado-Vivar, Sonia Zapata, Jéssica Duchicela, Pieter van ‘t Hof

**Affiliations:** ^1^ Department of Biological and Environmental Sciences - COCIBA, Universidad San Francisco de Quito-USFQ, Quito, Ecuador; ^2^ Institute of Microbiology, Universidad San Francisco de Quito-USFQ, Quito, Ecuador; ^3^ Tiputini Biodiversity Station, Department of Biological and Environmental Sciences - COCIBA, Universidad San Francisco de Quito-USFQ, Quito, Ecuador; ^4^ Department of Life Sciences and Agriculture, Universidad de las Fuerzas Armadas-ESPE, Sangolquí, Ecuador

**Keywords:** mycobiome, *Inga* (Fabaceae), arbuscular mycorrhizal fungi (AMF), Glomeromycotina, Amazon rainforest, Ecuador

## Abstract

This study explored the composition of the mycobiome in the rhizosphere of *Inga* seedlings in two different but neighboring forest ecosystems in the undisturbed tropical Amazon rainforest at the Tiputini Biodiversity Station in Ecuador. In terra firme plots, which were situated higher up and therefore typically outside of the influence of river floods, and in várzea plots, the lower part of the forest located near the riverbanks and therefore seasonally flooded, tree seedlings of the genus *Inga* were randomly collected and measured, and the rhizosphere soils surrounding the root systems was collected. Members of the Fabaceae family and the genus *Inga* were highly abundant in both forest ecosystems. *Inga* sp. seedlings collected in terra firme showed a lower shoot to root ratio compared to seedlings that were collected in várzea, suggesting that *Inga* seedlings which germinated in várzea soils could invest more resources in vegetative growth with shorter roots. Results of the physical-chemical properties of soil samples indicated higher proportions of N, Mo, and V in terra firme soils, whereas várzea soils present higher concentrations of all other macro- and micronutrients, which confirmed the nutrient deposition effect of seasonal flooding by the nearby river. ITS metabarcoding was used to explore the mycobiome associated with roots of the genus *Inga*. Bioinformatic analysis was performed using Qiime 2 to calculate the alpha and beta diversity, species taxonomy and the differential abundance of fungi and arbuscular mycorrhizal fungi. The fungal community represented 75% of the total ITS ASVs, and although present in all samples, the subphylum Glomeromycotina represented 1.42% of all ITS ASVs with annotations to 13 distinct families, including Glomeraceae (72,23%), Gigasporaceae (0,57%), Acaulosporaceae (0,49%). AMF spores of these three AMF families were morphologically identified by microscopy. Results of this study indicate that AMF surround the rhizosphere of *Inga* seedlings in relatively low proportions compared to other fungal groups but present in both terra firme and várzea Neotropical ecosystems.

## 1 Introduction

The Amazon region extends 8 million km^2^ of intact tropical rainforest where the Amazon River and its tributaries transport approximately 20% of the Earth’s freshwater, from the Andes, crossing nine countries in South America all the way down to the Atlantic Ocean. The Amazon is estimated to comprise 50% of the remaining global tropical forests on our planet and is considered a biodiversity hotspot with endemic and endangered flora and fauna (Guayasamin et al., 2021). The Amazon rainforest can be divided in 4 main forest habitats: non-flooded terra firme forests which dominate the Amazon region, followed by large extensions of várzea which are seasonally flooded by white water rivers. Seasonally flooded forest next to black water rivers are called igapó, and natural areas with white sandy soils are known as campina, but both have smaller distributions (Ritter et al., 2019). Várzea forests are rich in eroded sediments resulting in more fertile soils as they are seasonally flooded from rivers that originate high up in the Andes, and typically host fewer tree species compared to non-flooded terra firme forests (Wittmann et al., 2006). The elevated terra firme forests have relatively well-drained soils, and their vegetation structure is commonly divided by strata, whereas várzea forests present a deeper layer of organic matter, with less clay content, and a higher capacity to capture soil moisture in comparison to terra firme sites (Ensley-Field, 2016). Although in the Amazon rainforest the nutrient cycle is incredibly fast, yet the bioavailability of essential nutrients like phosphorus (P) and nitrogen (N) is generally low. Although high temperature and precipitation in Neotropical forests promote leaf decomposition and subsequent nutrient release, over time, lixiviation will leach nutrients to deeper layers where plant roots encounter difficulties absorbing those nutrients.

To explore the concept of nutrient availability in tropical ecosystems, and to comprehend how the Amazon soils can sustain the highest plant diversity on the planet despite low P quantities, requires a thorough understanding of soil characteristics and biogeochemical processes, and the role microorganisms play within these processes (Ensley-Field, 2016). Tropical ecosystems like the Amazon provide the broadest ecological niche for the development of microbial interactions, and it is thought that plant-associated soil fungi play a key role in promoting and maintaining this high plant diversity in tropical ecosystems (Bennett et al., 2017; Schappe et al., 2017; Sheldrake et al., 2017). One of the most important terrestrial mutualistic relationships are associations between mycorrhizal fungi and approximately 85% of all vascular plants (Van Der Heijden et al., 2017; Brundrett and Tedersoo, 2018; Field and Pressel, 2018). Specifically, the arbuscular mycorrhizal fungi (AMF) are considered the most diverse and widespread fungal partners of land plants, belonging to a group of specialized fungi that colonize and inhabit the rhizosphere (Smith and Read, 2008; Strullu-Derrien et al., 2018). For more than 450 million years, the coevolution between terrestrial plants and AMF has provided an important selective advantage for both partners and is even thought to have facilitated the earliest plant colonization of terrestrial ecosystems (Smith and Smith, 2011; Tedersoo et al., 2017; Field and Pressel, 2018; Vasco-Palacios et al., 2018). AMF are obligate mutualistic fungi, with around 50,000 species known to form associations with over 250,000 vascular and non-vascular plants in all terrestrial ecosystems (Chen et al., 2018). This wide distribution points towards crucial roles in the maintenance and structure of global plant communities (Walder et al., 2012; Marinho et al., 2018).

AMF are aseptate filamentous fungi which hyphae enter the root, where they construct their mycelium in the intercellular apoplastic spaces, until they build intracellular fungal structures called “arbuscules”, which serves as an interface for the exchange of nutrients (Field and Pressel, 2018; Strullu-Derrien et al., 2018). The bidirectional exchange of fungal-acquired nutrients like P and N through the fungal periarbuscular membrane for plant-fixed carbon (C) is an integral part of the AMF mutualism (Schappe et al., 2017; Field and Pressel, 2018). Up to 20% of the plant photosynthates are transferred from the host plants to AMF, essential to complete their biological cycle (Tang et al., 2016). In return for carbon-based compounds, AMF transfer the equivalent of up to 90% of the phosphorus and 80% of the nitrogen requirements for plant growth and development (Smith and Read, 2008; Marinho et al., 2018). Although this exchange is considered a costly investment for the host plant, it is through this reciprocal exchange that both partners can meet their important physiological needs. Physical-chemical soil properties on the one hand, and edaphic factors like soil pH, water content, acidity, and soil aeration on the other hand determine AMF species richness and community structure (Wang et al., 1993; de Oliveira Freitas et al., 2014; Lugo and Pagano, 2019).

AMF perform a variety of ecosystem services, as their hyphal networks physically extend the plant’s rhizosphere, allowing plants to access a larger soil volume to promote the overall absorption of water and nutrients (Marinho et al., 2018). At a local level, AMF associations shape the plant community composition and overall plant productivity, promoting plant fitness and boosting reproductive capacities (Asghari and Cavagnaro, 2011; Cofré et al., 2019; Tedersoo et al., 2020). But mycorrhizal partnerships also play an important role in the global carbon cycle by augmenting soil C sequestration processes (Averill et al., 2014; Van Der Heijden et al., 2017; Field and Pressel, 2018).

Although the arbuscular mycorrhizae type is the most studied plant-fungus association, there has been a bias towards AMF research in temperate regions, with considerably less AMF research being conducted in the tropics (Cofré et al., 2019; Peña-Venegas and Vasco-Palacios, 2019). AMF communities in primary tropical forest ecosystems can be complex, and so are the factors that drive the community establishment. Moreover, the proportion of mycorrhizal plants increases towards the equator, which may reflect the arbuscular mycorrhizal ancestral state and tropical origin of major plant clades (Delavaux et al., 2019). To generate a better understanding of these aspects at an ecological level, there is a need to generate greater knowledge about this group at a taxonomic level and their specific ecological and evolutionary importance (de Oliveira Freitas et al., 2014).

For a long time, it has been a generally accepted dogma that plants in tropical rainforest ecosystems, with their exceptionally high turnover of nutrients and optimal climatic conditions around the equator, simply do not need to associate themselves with beneficial fungi. However, in recent years, there is growing evidence that AMF diversity in fact is high in tropical ecosystems (Peña-Venegas and Vasco-Palacios, 2019). As previously mentioned, many Neotropical rainforest soils in fact feature P deficiencies, and there might be an increased selective pressure on tropical plants to invest in the establishment of AMF associations. According to Marinho et al. (2018), tropical forests harbor 75% of all Glomeromycotina species known to date and might therefore be considered hotspots for AMF diversity. Furthermore, it has been proposed that the highest number of undescribed fungal species could be found in tropical biodiversity hot spots (Lugo and Pagano, 2019).

Given this extraordinary prediction about the AMF diversity in South America, there is a great potential to explore AMF in the Amazon rainforest. Although classified as one of the world’s important biodiversity hotspots regarding the macrobiodiversity, it could consequently be considered an important region to study the microbiodiversity that functionally supports the visible flora and fauna. This microbial biodiversity has been relatively well described for the Amazon regions of Colombia, Brazil, Venezuela, and Peru, whereas many microorganisms, including bacteria and fungi, but also insects are still largely unknown for the Amazon rainforest of Ecuador (Cañarte et al., 2020). Our research team recently published that the mycorrhizal diversity as mentioned in literature studies at the Ecuadorian Amazon is similar to other studies in the Amazon region but concluded that the number of undescribed mycorrhizal species in the tropical forests of Ecuador need more attention as patterns of occurrence and diversity remain largely unknown (Duchicela et al., 2022).

The present study investigated the fungal abundance and diversity in terra firme and várzea soils associated to the roots of tree seedlings of the genus *Inga*, an exclusively Neotropical genus belonging to the Fabaceae, a diverse family known to invest in associations with beneficial bacteria and fungi (de Oliveira Freitas et al., 2014; Antunes et al., 2019). Members of the genus *Inga* are omnipresent in both forest ecosystems within the study area at the Tiputini Biodiversity Station (TBS), a pristine and undisturbed tropical research area situated in the Yasuní Biosphere Reserve in the Orellana Province of Ecuador. We measured the roots and shoots of *Inga* seedlings and collected rhizosphere soil samples to measure soil physical and chemical properties, and to extract DNA to perform metabarcoding methods to assess what fungi, with a special focus on AMF, were present in the rhizosphere of *Inga* sp. Moreover, we were interested whether the abundance and composition of the *Inga* rhizosphere mycobiome differed between terra firme and várzea sampling sites, and what factors could influence these possible differences. Furthermore, we used microscopy to morphologically identify AMF spores to compare them with the metabarcoding results. Considering the differences in forest structure and abiotic factors within each ecosystem, we expected to find AMF to be present in both ecosystems, but the AMF community structure to differ in response to these conditions. Hence, in terra firme forests, where soils tend to be poorer in nutrients in comparison to várzea soils, a higher degree of AMF colonization is expected, and possibly a higher abundance and diversity of AMF in close proximity to the roots of *Inga* seedlings.

## 2 Methodology

### 2.1 Study area description

**Figure 1:**
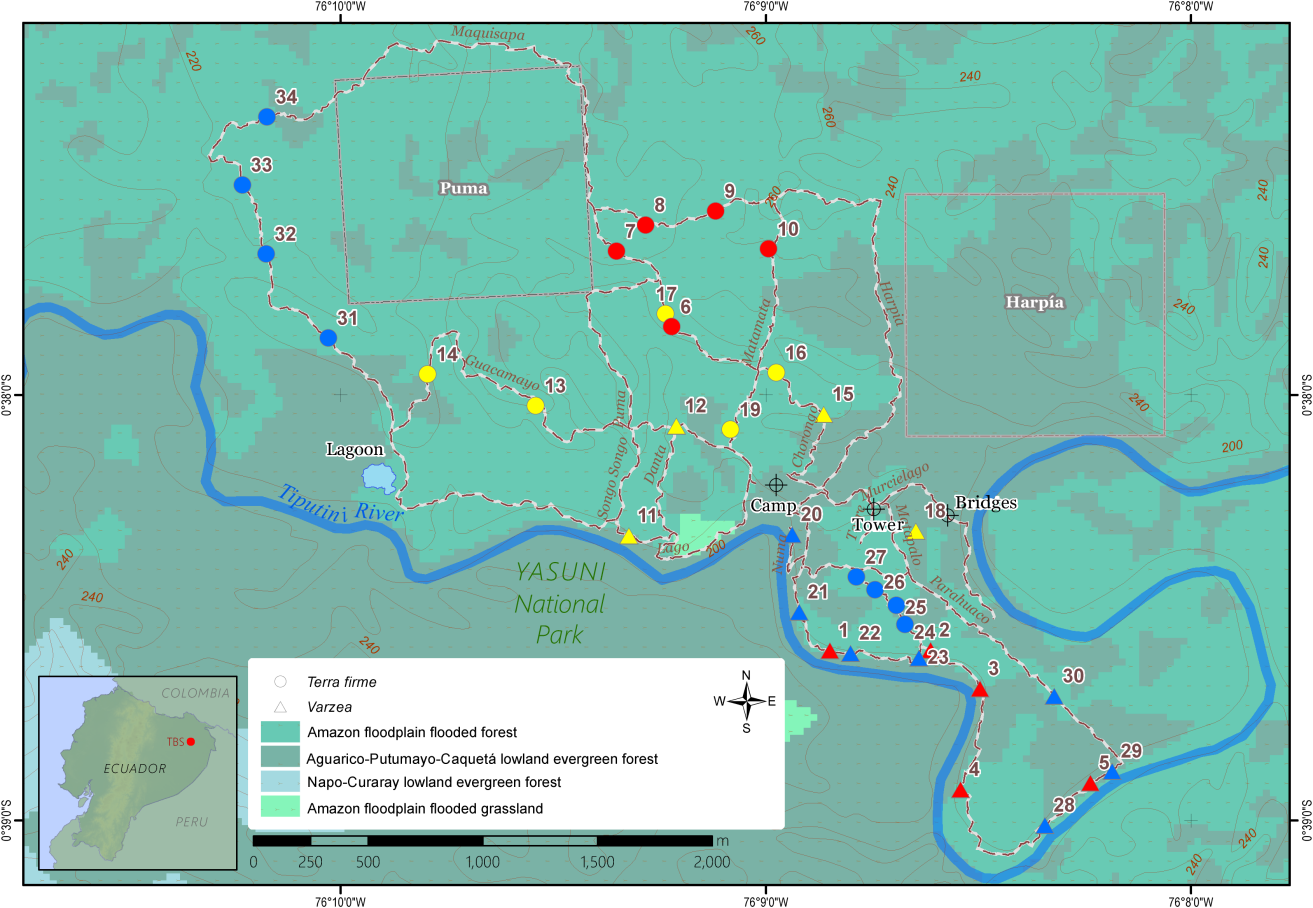
Map of the sampling locations for *Inga* seedling in the Tiputini Biodiversity Station in the Ecuadorian Amazon rainforest. Station trails are represented by intercepted white-brown lines, while the various ecosystem types are depicted in a gradient of green background colors. Triangle symbols *Inga* collections in várzea; Circles indicate sampling points in terra firme. Colors indicate different sampling expedition: red, November 2018; yellow, August 2019; blue, February 2020. The green to blue color gradient indicates the ecosystem classification according to the Ecosystem Classification System of Continental Ecuador. Source: Ministerio del Ambiente del Ecuador (2013).

The current study took place at the Tiputini Biodiversity Station (TBS), situated within a pristine area of primary Amazon rainforest with minimum human impacts in the province of Orellana, Ecuador (**Figure 1**). This biological research station, which is located ca. 280 km ESE from Quito (00°37’05” S, 76°10’19” W, 270 m. above sea level), was established in 1994 by Universidad San Francisco de Quito (USFQ) in collaboration with Boston University (USA). With its fluvial source high up the flanks of several volcanoes in the Eastern part of the Andes, the Tiputini river, which downstream joins the Napo River and becomes one of the tributaries of the Amazon, separates the TBS on its northern bank inside the UNESCO Yasuní Biosphere Reserve, from the border of the Yasuní National Park, which starts on its Southern riverbank (Cisneros-Heredia, 2006). The Yasuní Biosphere Reserve, with an extension of 9,820 km^2^, occupies a unique location at the intersection of the Andes and the Amazon. Yasuní’s climate is characterized by relatively high temperatures (24–27°C, year-round), and a high relative humidity of 80–94% throughout the year (Bass et al., 2010). Meteorological data as logged by the TBS Vantage Pro 2 weather station (Davis Instruments Corporation, California, USA) were used to reconstruct the monthly temperature and precipitation regime during the timeframe of the sampling expeditions (**Supplementary Figure 1**). The Yasuní Biosphere Reserve is considered part of the Core Amazon region and has a year-round high annual rainfall which leads to the absence of a severe dry season. The soils of this area are geologically young, created by fluvial sediments derived from the weathering and erosion of the sources upstream in the Andes (Bass et al., 2010). The TBS has a total extension of 638 hectares of forests that can be divided in lowland evergreen forest or terra firme (~90%), and approximately 13% of its territory consists of periodically flooded várzea (or lowland evergreen forest seasonally flooded by white water rivers). Furthermore, the TBS has some small patches of igapo forest (lowland evergreen forest flooded by black water rivers).

### 2.2 Ecosystems and sampling design

Within the TBS research station, during three fieldwork expeditions between November 2018 and February 2020, tree seedlings of the genus *Inga* of várzea and terra firme forests were sampled. Sampling collection details are shown in **Table 1**, while sampling point coordinates are displayed in the TBS map in **Figure 1**. In várzea forests, less ground cover with a low abundance of *Inga* seedlings was observed, particularly in areas with visual evidence of recent flooding. Areas closer to the Tiputini river presented distinct physical and biological structures related to the influence of seasonal floods, such as the presence of aerial roots, general suppression of plant growth and the formation of erosion slides. Within established TBS 1 ha plots, the total number of subadult and adult trees in terra firme plots (737 individuals) and várzea (714 individuals) have been assessed from 1998 until present. These numbers were used to calculate local relative abundances on the family (Fabaceae) and genus (*Inga*) level within terra firme and várzea forest plots. The total abundance of Fabaceae trees within terra firme plots of the TBS was 13.2%, with almost half of these trees belonging to the genus *Inga* (5.7%). Within várzea forests, 25.4% of all subadult and adult trees encountered in the vegetation belonged to the Fabaceae family, of which more of half of the individuals (14.2%) could be identified as *Inga* sp. (personal communication Gonzalo Rivas-Torres). Although the distribution of seedlings of *Inga* sp. was not homogenous along the station trails, in general it was not difficult to encounter them.

**Table 1:** Overview of the sample collection at the Tiputini Biodiversity Station during three sampling campaigns.

Sample type	Objective	Sample # in várzea	Sample # in terra firme	Field campaign
*Inga* soil rizosphere	Metabarcoding	9	10	Nov-18 & Aug-19
*Inga* soil rizosphere	Physical-chemical soil analysis	5	5	Nov-18
*Inga* plantlets	Shoot/roots measurements	39	24	Feb-20
*Inga* soil rizosphere	AMF spore identification	8	7	Feb-20

### 2.3 Physical and chemical soil properties

Soil samples were collected from the rhizosphere of *Inga* sp. seedlings in várzea and terra firme forests. During sampling, *Inga* sp. seedlings where excavated, and soil clumps attached to the seedling root structure were collected for physical and chemical soil analysis. To take a more representative sample soil, it was collected from varying depths, between 5 cm to 30 cm from the soil surface. 250g of soil was stored in 50 ml Falcon tubes. Soil samples were transported to the USFQ campus and stored at 4 °C. Samples where dried and sieved before processing at the laboratory facilities of the USFQ Environmental Engineering Department. Soil samples were characterized using the EPA 3050B method for initial sample mineralization and EPA 6010B for instrumental measurement. Soil analysis was conducted using a plasma spectrophotometer (Thermo Scientific brand, iCap 7000 model) with inductive coupling and optical detector (ICP-OES). Certipur^®^ standards were used for instrument calibration, with an ICP multi-element standard solution VIII (Merck-Millipore, Switzerland). For quality control in recoveries, estuarine sediment as the Standard Reference Material (SRM^®^) from NIST^®^ number 1646 was used (SigmaAldrich/Merck, Switzerland).

### 2.4 Phenotypic characterization *Inga* seedlings

The *Inga* genus is characterized by the presence of winged rachis, a morphological characteristic that permits easy identification in the field (Heywood et al., 1993). At 39 várzea sites and 24 terra firme sites alongside a multitude of TBS trails, *Inga* sp. seedlings were spotted, photographed, GPS referenced, and for each seedling, root and stem length were measured using ImageJ software (Schneider et al., 2012).

### 2.5 Molecular analysis of rhizosphere samples of *Inga* sp.

The soil rhizosphere of 19 *Inga* seedlings was sampled from terra firme and várzea forest sites at the Tiputini Biodiversity Station (TBS) during two sampling expeditions in November 2018 and August 2019. Soil aggregates attached to the roots system were collected and stored in 1.5 ml Eppendorf tubes with LifeGuard Soil Preservation solution. Rhizosphere samples were transported to USFQ and stored at -20°C. The total DNA of the rhizosphere samples (< 2g of soil) was extracted by lysis with mechanical spheres. DNA purification was performed using the DNeasy PowerSoil kit following the manufacturer’s instructions (Qiagen, USA). Extracted rhizosphere DNA samples were shipped to Baseclear (Leiden, The Netherlands) for metabarcoding using the Internal Transcribed Spacer (regions ITS1 and ITS2) as a molecular marker (Nilsson et al., 2015). Sequencing was performed using the Illumina MiSeq platform (Read length was 2x300; PhiX 20% was used as sequencing control). Raw DNA sequences were analyzed using the bioinformatic package Qiime 2 version 2020.6 (Caporaso et al., 2011). Metadata were constructed including information for the variables sampleID, ecosystem (terra firme or várzea), year (2018 or 2019) and month (November or August). Keemei add-on in google sheets was used to validate the sample metadata (Ram et al., 2016). Denoising was carried out using the package DADA2 version 2017.2.0. (Callahan et al., 2016) using *trim-left=10* and *trunc-len=290* to perform a quality control of the demultiplexed sequences. The output artifacts were feature table (ASV table), a representative sequences table, and a statistical table (Caporaso et al., 2011).

Diversity analyses required a rooted phylogenetic tree which was generated using Qiime 2 phylogeny *align-to-tree-mafft-fasttree* pipeline to support Faith, weighted and unweighted unifrac diversity metrics. The α and β diversity were analyzed applying the Qiime 2 diversity core-metrics-phylogenetic method, with a sampling depth of 5500 to rarefy the samples (Caporaso et al., 2011). The feature table was filtered using the command *qiime taxa filter-table* to conserve both Fungi and Glomeromycota ASVs (new nomenclature: Glomeromycotina). The sampling depth for Fungi ASVs was 10000 and 96 for Glomeromycotina. Alpha diversity was calculated using Shannon’s diversity index, Observed Features, Faith’s Phylogenetic Diversity and Evenness. Statistical tests were performed and we used the Kruskal-Wallis test (Kruskal and Wallis, 1952) or PERMANOVA (Anderson, 2001) with a p-value < 0.05.

To assign the taxonomic classification of the ASV table, using the *q2-feature-classifier* plugin, using the database SILVA version 1324 (Bokulich et al., 2018). The taxonomic composition was viewed generating taxa bar plots using QIIME 2 taxa barplot commands. The ASV feature table was filtered using the q2-taxa plugin within the command *QIIME taxa filter-table* to include sequences only from Fungi kingdom and Glomeromycota phylum (Caporaso et al., 2011), although the name has been changed to Glomeromycotina and based on nuclear genomic data is currently recognized as a subphylum within Mucoromycota equally distant from the two other subphyla, Mucoromycotina and Mortierellomycotina (Spatafora et al., 2016; Bruns et al., 2018). We used ANCOM and Gneiss to identify features that were differentially abundant across groups (Caporaso et al., 2011). Finally, biplots were created using the *qiime gneiss balance-taxonomy* command (Morton et al., 2017).

### 2.6 Morphological characterization of AMF spores

A total of 15 soil samples from the rhizosphere of *Inga* seedlings were collected for AMF spore identification in the sites of study in February 2020. Soils were sampled to a depth of 15 cm, placed in plastic bags, dried in an incubator at 65°C for 24 hours, and stored at 4°C in a dry environment until shipment for processing at Universidad de las Fuerzas Armadas - ESPE. AMF spores were extracted by wet sieving and decanting (Gerdemann and Nicolson, 1963) followed by a 60% sucrose centrifugation (Sylvia et al., 2007).

Spores were cleaned and collected manually using an extruded 9-inch glass pipette, and separated by morphotypes based on spore color, size, and type of spore formation (acaulosporoid, glomoid, or gigasporoid) and mounted in polyvinyl alcohol lacto glycerol (PVLG) (Koske and Tessier, 1983) and PVLG mixed with Melzer’s reagent (1:1 v/v) for microscopic examination. Spore color was determined by comparing spores to a printed color chart proposed by the International Culture Collection of (Vesicular) Arbuscular Mycorrhizal Fungi (INVAM). Morphological identification was carried out by observing spore wall characters (*e*.*g*., wall thickness, ornamentations, Melzer’s reaction), the terminology used to describe subcellular spore wall structure is based on the spore development model as proposed by Morton et al. (2017). Comparisons with descriptions of AMF reference cultures of INVAM were made (https://invam.ku.edu/). Nomenclature for AMF genera and classification follows Redecker et al. (2013).

### 2.7 PCoA analysis

To display the differences in the fungal ASV composition as present in 3 terra firme and 3 várzea samples as collected from the rhizosphere of *Inga* tree seedlings, a principal component analysis (PCoA) was performed to construct a 2D graph to visualize correlations between the relative abundance information of the taxonomic composition of fungal ASVs and all chemical properties measured as previously described. The larger the distance between two coordinates in the PCoA graph, the less similar they are. We used the ‘envfit’ function of the vegan R package to calculate the environmental vectors, and the PCoA plot was created using the ‘ggplot2’ package (R version 4.2.2).

## 3 Results

### 3.1 Physical-chemical soil properties of várzea and terra firme soils

During each field expedition, sample site descriptions were conducted to evaluate visible soil characteristics, and to assess possible differences between sample sites. It was observed that in the terra firme plots, leaf litter and humus accumulation was visibly higher, less compacted, and less degraded. In várzea forest sites, leaf litter and humus accumulation in these soils had a darker colour, tended to be waterlogged, and was more compressed into a thinner layer. Macro- and micronutrients with important roles in plant development were evaluated for soil samples collected at várzea and terra firme sites at the TBS, and presented a normal distribution, which was confirmed using a Kolmogorov-Smirnov test (**Supplementary Table 1**). All samples were found to be below the critical K-S value of 0.41. Potassium (K), zinc (Zn), iron (Fe), aluminum (Al), lead (Pb), nickel (Ni), chromium (Cr), cadmium (Cd), pH values, and soil conductivity did not present significant differences between the ecosystems (**Supplementary Table 1**, labelled “ns”). All other analysed physical and chemical soil parameters showed significant differences between soil samples collected from várzea and terra firme sites. Soil samples collected in forest plots of terra firme resulted in a higher nitrogen (N) proportion (t(8) =0.024, p = 0.05) compared to soils collected in várzea forest sites. Although phosphorous (P) was found to differ slightly between ecosystems (t(8)=0.043, p=0.05), a higher concentration of this macronutrient was found in várzea forests compared to terra firme. Magnesium (Mg) and calcium (Ca) showed a higher statistical significance, with p values equal or below 0.01, and both were found in higher concentrations in várzea forests. Molybdenum (Mo) and manganese (Mn) showed the highest differences between the two ecosystems. Similar trends could be observed for all other micronutrients, with higher average concentrations in soil samples for várzea forest plots. Furthermore, várzea soils were found to be slightly less acidic than in terra firme. Moreover, várzea soils resulted in significantly higher sodium (Na) concentrations, which might indicate a higher concentration of sodium salts, compared to terra firme soils. Cobalt (Co) (t(8)=6.897E^-06^, p=0.05), presented the highest statistical difference for all elements measured and was found at considerably higher concentrations for soil samples collected in várzea, than in terra firme. In contrast, vanadium (V) (t(8)=0.026, p=0.05), presented higher concentration in terra firme forests than in várzea forests.

### 3.2 Root and shoot measurements of *Inga* seedlings in várzea and terra firme

**Figure 2:**
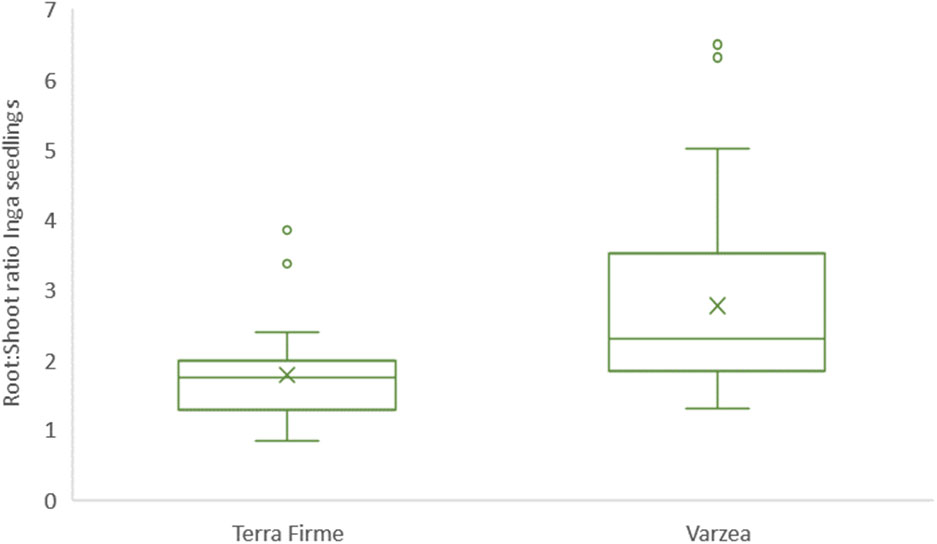
Boxplots indicating shoot:root ratio of *Inga* sp. seedlings collected in terra firme and várzea forest sites. Seedlings were collected during February 2020 at the Tiputini Biodiversity Station in Ecuador. In each box plot, data is plotted as the median (midline) and percentiles (box, 25th and 75th percentiles), the whiskers and individual points (outliers) represent all values within and above 1.5 times the interquartile range, respectively. Additionally, means were plotted as “X”.

Both root and shoot lengths were measured for 63 randomly sampled seedlings of *Inga* sp. with a comparable developmental stage using ImageJ software. Root and shoot lengths proved not to be statistically different, but certain biological trends were observed within each tropical forest ecosystem: On the one hand, roots of *Inga* seedlings collected in várzea sites had the tendency to be shorter (9.84 cm ± 3.16 cm) than those collected in terra firme sites (10.66 cm ± 3.7 cm). On the other hand, shoots of *Inga* seedlings collected in várzea plots had the tendency to be longer (23.54 cm ± 7.41 cm), than those sampled in terra firme plots (17.73 cm ± 3.99 cm). To further explore the relationship between shoot and root lengths between ecosystems, a shoot and root ratio was calculated (**Figure 2**). This ratio was obtained by dividing shoot length by root length for each *Inga* seedling sampled. Average shoot to root ratio for *Inga* individuals sampled in terra firme plots was 1.78 ± 0.70, whereas this ratio for *Inga* seedlings collected in várzea plots was 2.77 ± 1.28.

### 3.3 Fungal and AMF abundance and diversity in the rhizosphere mycobiome of *Inga* sp.

**Figure 3:**
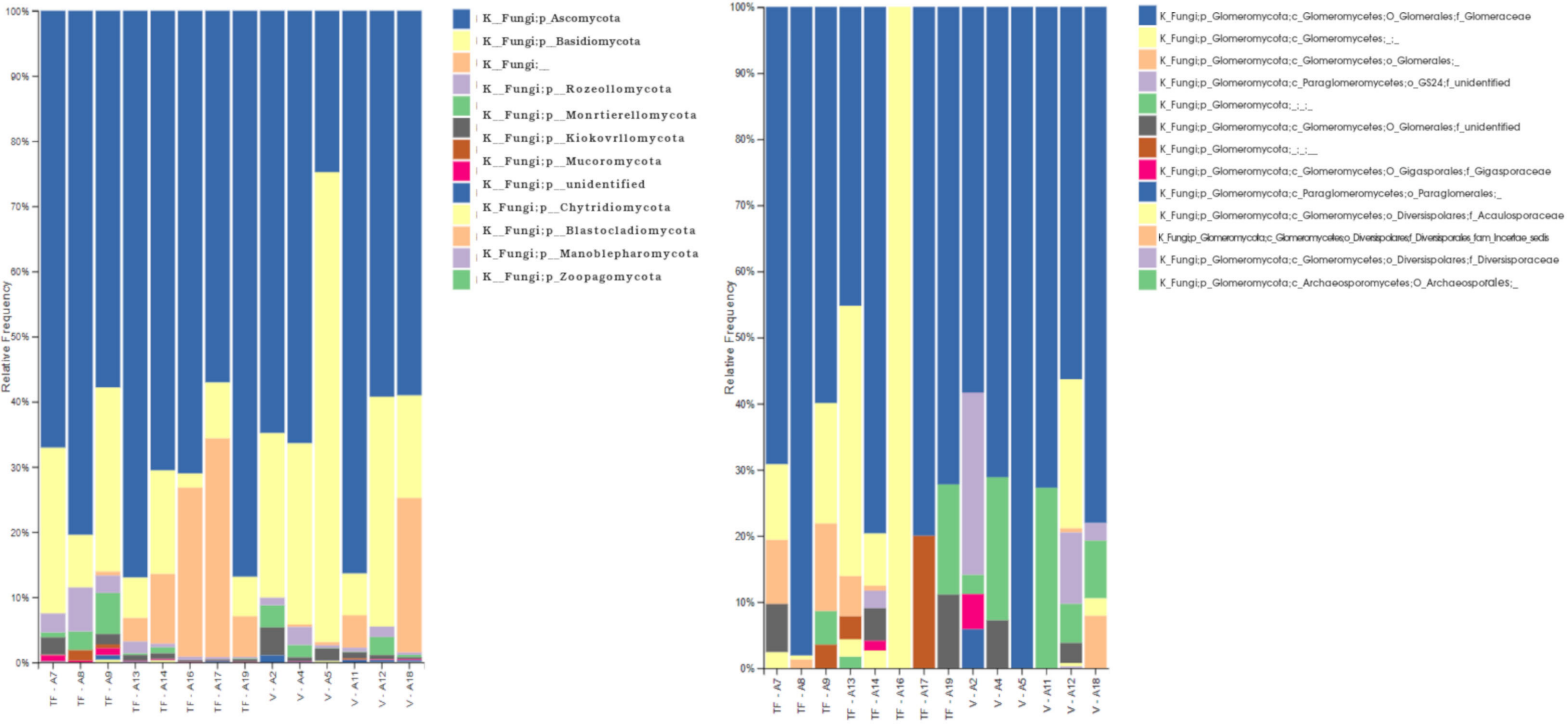
Results of ITS amplicon sequencing of rhizosphere soil samples of *Inga* seedlings collected in várzea (V) and terra firma (TF) ecosystems at the Tiputini Biodiversity Station in the pristine Ecuadorian Amazon. ITS sequences were analyzed using Qiime2. Left panel bar plot representing specific ASVs annotated to the Fungi kingdom; Right panel barplot representing ASVs belonging to the subphylum Glomeromycotina (formerly known as the phylum Glomeromycota).

To explore the rhizosphere mycobiome of *Inga* sp., a total of nineteen samples were analyzed from várzea (n=9) and terra firme (n=10) forest ecosystems. Ten samples were collected in November 2018, and 9 samples in August 2019. The ITS metabarcoding results showed a total of 1 207 868 raw sequences (Paired End Sequences), with 5776 identified amplicon sequence variants (ASVs) using DADA2. The median of the quality score of the data was 30. Five samples were excluded from the analysis, due to the low numbers of features, which established 8 rhizosphere samples from terra firme, and 6 samples from várzea ecosystems. Taxonomic analysis was performed to identify the rhizosphere mycobiome composition, with ITS ASVs annotate to 10 kingdoms, of which Fungi was dominant (77,52%), followed by an unidentified kingdom (16,9%), Viridiplantae (3,58%), Alveolata (1,2%). The remaining 0,8% corresponded to other minor kingdoms (**Supplementary Figure 2**). The feature table was filtered to analyze the Fungal composition, resulting in 404 968 ASVs. Eleven phyla were identified and two were unidentified: Ascomycota (68,67%), Basidiomycota (18,59%), Rozellomycota (2,06%), and the Glomeromycota (now subphylum Glomeromycotina) (1,42%). The remaining 9,26% corresponded to other minor phyla (**Figure 3** – left panel). The subphylum Glomeromycotina (formerly known as the Phylum Glomeromycota) was represented by 5 747 ASVs, with a total of 13 families (5 previously described families, and 8 unidentified): Glomeraceae (72,23%), Gigasporaceae (0,57%), Acaulosporaceae (0,49%), Diversisporales (0,16%), Diversisporaceae (0,03%), the remaining 26,52% correspond to ASVs that could not be annotated to a specific family but nevertheless were annotated as AMF (**Figure 3** – right panel). The general richness of the rhizosphere mycobiome of *Inga* plantlets based on α-diversity was calculated using Evenness, Faith and Shannon’s indexes, and was not significantly different (p>0,05). For the Fungi kingdom, evenness and Shannon indexes across collection dates were significantly different (p=0,01 and p=0,04, respectively). However, the richness across ecosystems was not significantly different (p>0,05). Finally, the α-diversity within the identified Glomeromycotina did not reveal clear composition differences neither between ecosystems nor collection dates (**Supplementary Table 2**).

**Figure 4:**
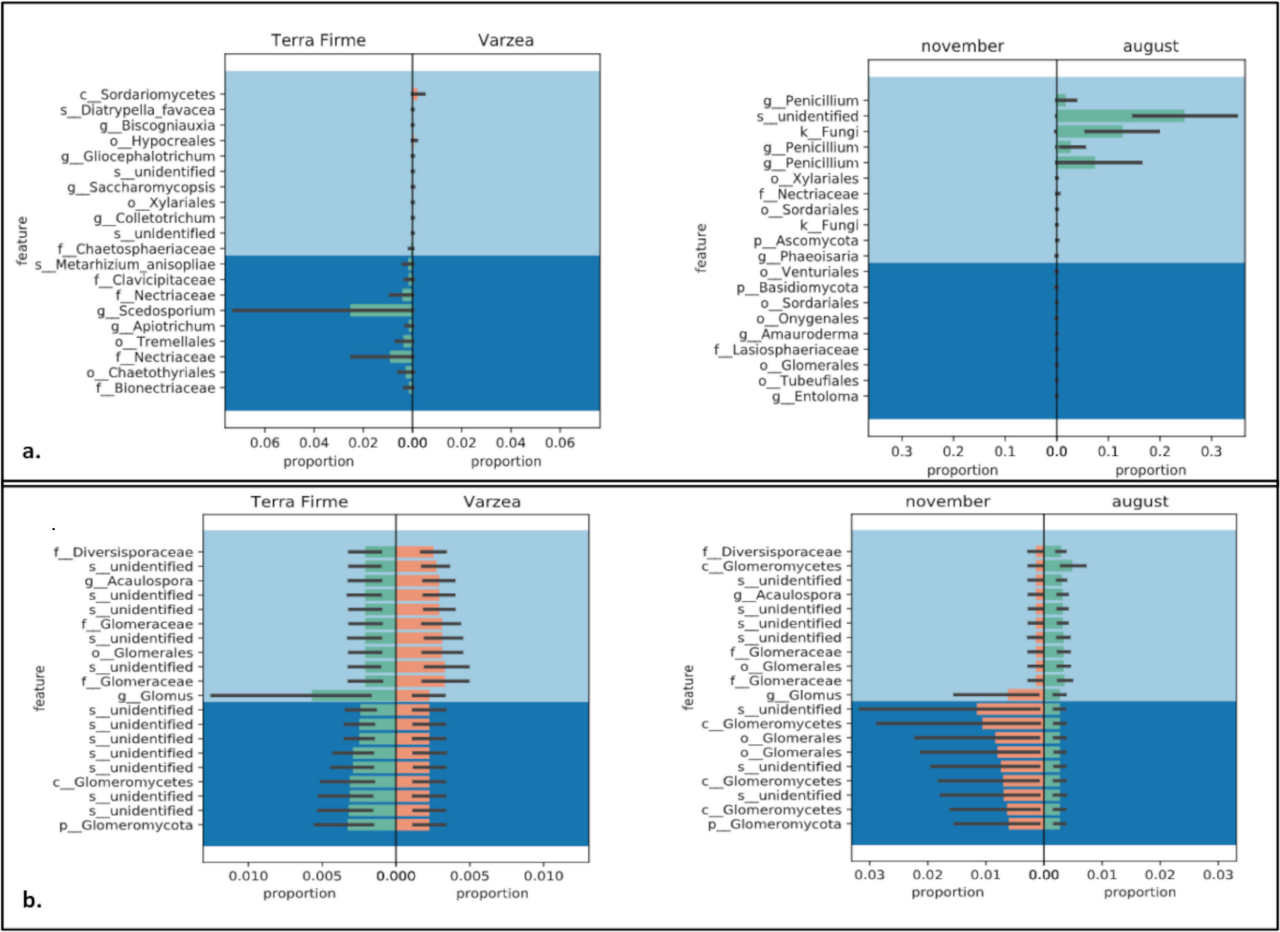
Comparison of abundances of fungal and Glomeromycotina taxa analyzed by Gneiss metrics. Panels show differentially abundant Fungi **(A)** and Glomeromycotina **(B)** associated to the rhizosphere of *Inga* seedlings collected in terra firme or várzea forest ecosystems (left panels) or sampled in November 2018 and August 2019 (right panel). Taxons are described to the taxonomic most detailed annotation: P, phylum; c, class; o, order; f, family; g, genus; and s, species.

To explore the composition of the rhizosphere mycobiome structure, the β-diversity was calculated using Bray-Curtis, Jaccard, Weighted, and Unweighted UNIFRAC indexes (**Supplementary Table 2**). The Jaccard, Unweighted and Weighted UNIFRAC indexes indicated significant differences in the rhizosphere composition of *Inga* plantlets collected in terra firme compared to várzea (p=0,002, p=0,04 p=0,04, respectively). Moreover, the rhizosphere composition indicated a collection date effect, as both Bray Curtis and Weighted UNIFRAC PERMANOVA tests showed significant differences between samples collected in November 2018 compared to samples collected in August 2019 (p=0,002). Similar trends were observed analyzing only Fungi ASVs, where Jaccard and Unweighted UNIFRAC revealed significant differences between ecosystem (p=0,003 and p=0,03, respectively). Finally, the **β**-diversity calculations for ASVs annotated to the phylum Glomeromycota (now subphylum Glomeromycotina) did not show differences for sampling location nor sampling date (p>0,05). We used GNEISS metrics to calculate and identify differentially abundant fungal taxa by comparing ecosystems (terra firme vs várzea) and sampling date (November 2018 vs August 2019). When analyzing exclusively ASVs belonging to the kingdom Fungi, the most abundant taxon in várzea samples was annotated to the Sordariomycetes class, while in the terra firme samples the most abundant taxons were annotated to the genus *Scedosporium* and two members of the Nectriaceae family (**Figure 4A**). When comparing differential abundances between the samples collected in November 2018 and August 2019, it was found that three taxa of the genus Penicillium, one taxon of unidentified species, and one taxon annotated as a Fungi, were present in higher abundances in August 2019 (**Figure 4B**). When we used GNEISS metrics to explore whether there were differential abundances within the ASVs belonging to the Glomeromycotina, it was observed that members belonging to the genus *Glomus* were more abundant in terra firme compared to várzea, and that a taxon belonging to an unidentified species of Glomeromycotina and a taxon of the Glomeromycetes class were more abundant in November 2018 compared to August 2019 (**Figure 4**).

### 3.4 Morphological characterization of AMF spores

**Figure 5:**
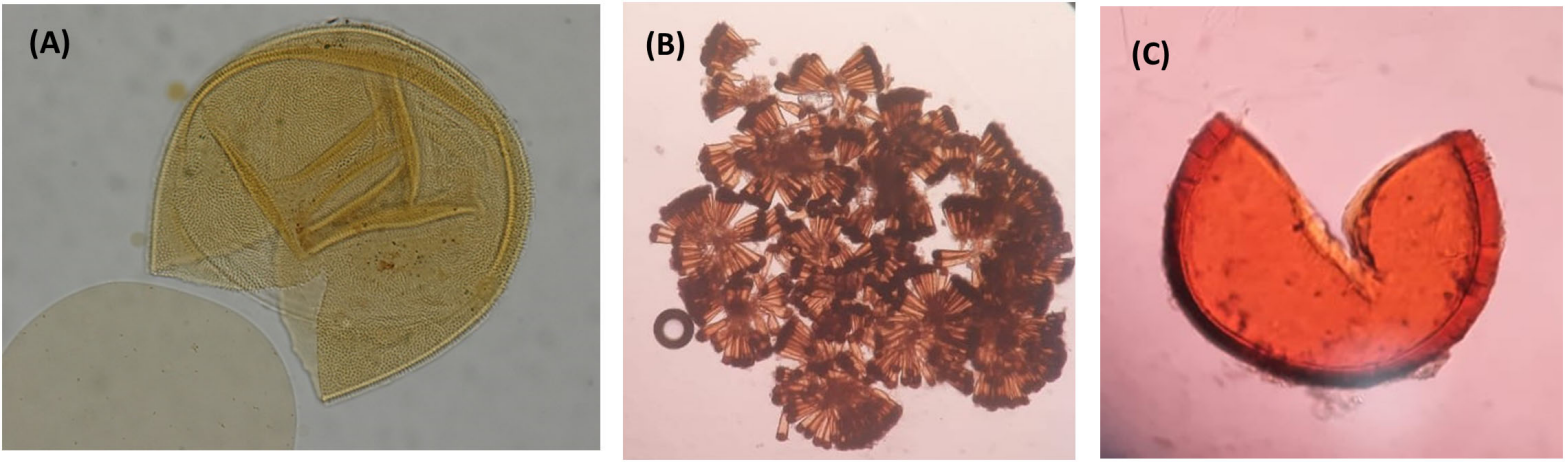
Morphological characterization of AMF spores present in the rhizosphere of seedlings of the genus *Inga* in the Ecuadorian Amazon. **(A)** Microscopic image of a spore of *Acaulospora spinosa;* **(B)** Microscopic photo of the remainings of a sporocap contributed to the genus *Sclerocystis* (*Glomus* sp.) **(C)** Photo of the characteristic spore characteristic for AMF belonging to *Gigaspora*.

After processing a total of fifteen rhizosphere samples from *Inga* seedlings collected at the TBS research station in February 2020, AMF spores were encountered in most of the rhizosphere samples. Using microscopy, the recovered spores could be attributed to three arbuscular mycorrhizal fungal families based on AMF spore morphology: Glomeraceae, Gigasporaceae, and Acaulosporaceae (**Figure 5**).

### 3.5 Associating mycobiome rhizosphere composition with environmental covariates

**Figure 6:**
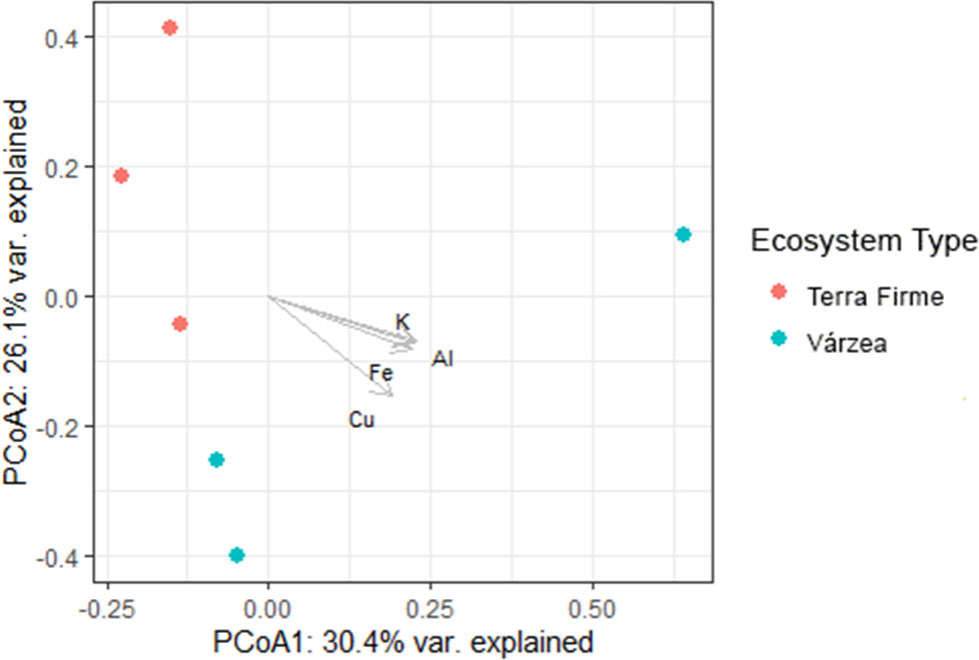
Principal Component Analysis (PCoA) correlating soil physical and chemical properties and mycobiome composition of the rhizosphere of *Inga* sp. seedlings collected at Tiputini Biodiversity Station in the Ecuadorian Amazon. Tropical forest ecosystems: red dots: terra firme, blue dots: várzea. The first and second canonical axis explain together 56.5% of the variation. The vectors indicate soil chemical properties which explain the highest amount of variation: K: potassium, Al: aluminium, Fe: Iron, Cu: copper.

The PCoA based on the relative ASVs abundance indicated that the rizosphere of *Inga* seedlings collected in terra firme or várzea forest plots established distinct fungal communities (**Figure 6**). The PCoA coordinates plot explained 56.5% of the total observed variation, where the first axis explained 30.4% of the variation, and the second axis explained 26.1%. The environmental vectors indicated the soil chemical elements K, Al, Fe, and Cu, to explain the highest amount of variation between the fungal rhizosphere communities of *Inga* in terra firme or várzea forest soils.

## 4 Discussion

This study explored the composition of the mycobiome in the rhizosphere of *Inga* seedlings in two different but neighboring forest ecosystems in the undisturbed tropical Amazon rainforest of the Tiputini Biodiversity Station in Ecuador. In terra firme plots, which were situated higher up and therefore typically outside of the influence of river floods, and in várzea plots, the lower part of the forest located near the riverbanks and therefore seasonally flooded, tree seedlings of the genus *Inga* were randomly collected and measured, and we sampled the rhizosphere soils surrounding the root systems. We used an array of techniques, including physical-chemical soil analysis, metabarcoding using the molecular marker ITS to characterize the fungal rhizosphere community structure, and microscopy to morphologically identify AMF spores.

Within the TBS, members of the Fabaceae family and specifically the genus *Inga* demonstrate a high abundance, in particular in várzea habitats where 14.2% of all adult and subadult trees were identified as members of the genus *Inga* (personal comment Gonzalo Rivas-Torres). A factor that could explain the high abundance of this group might be their ability to form rhizospheric associations with both nitrogen fixing bacteria and AMF. These plant-associated microorganisms promote plant health by positively stimulating plant growth, boosting physiological responses towards biotic and abiotic stress factors, and supporting the plant’s adaptation to environmental circumstances (Mendes et al., 2011).

Temperature is an environmental factor that can have major effects on both plant growth and root exudation, thereby directly influencing AMF vesicle formation and colonization (Graham et al., 1982; Fitter et al., 2004). Furthermore, studies have shown that AM fungi can also directly be affected by temperature (Hawkes et al., 2008). This stimulating effect of elevated temperature on AMF growth and inoculation could explain why in tropical ecosystems, characterized by high temperatures, there is a greater abundance and diversity of AM fungi. It is therefore expected that these climatic conditions provide a selective force on AMF populations (Hawkes et al., 2008). The average temperatures as logged by the TBS weather station during the timeframe of our study are consistent with the normal average annual temperature (25.1°C) and within the range of common seasonal changes in temperature (personal communication TBS STAFF). As our study is a baseline mycobiome exploration study and should be considered as the first one of its kind in the Yasuní National Park area in Eastern Ecuador, the current study design does not allow to correlation prevailing temperature regimes and possible shifts in the belowground mycobiome composition. However, the period January-March 2020 was considerably drier compared to the same period in 2018 and 2019, which is directly correlated with the local flooding incidence. It has been reported that roots of plants in flooded soils present a lower AM fungi diversity (Deepika and Kothamasi, 2015). In tropical ecosystems like the current study site (TBS), this parameter could therefore function as an environmental filter, which might result in a reduced number of AMF species which can tolerate these prevailing habitat conditions, due to low prevailing oxygen levels and soil nutrient inputs during floods. but these conditions could put evolutionary pressure towards flood-tolerant AMF phylotypes, specifically evolved to survive extended periods with low oxygen contents in the soils during such a flood

As a bioindicator whether *Inga* seedlings in terra firme and várzea invest more of their resources aboveground or belowground, we measured the shoots and primary roots of randomly sampled *Inga* tree seedlings. The shoot:root ratio of *Inga* seedlings growing in terra firme plots had the tendency to present lower ratios compared to *Inga* seedlings developing in várzea plots (**Figure 4**). Hence, *Inga* tree seedlings seemed to use relatively more of their acquired nutritional energy in the development of their root systems in terra firme, compared to várzea, where they invested more in vegetative growth. Although the tendencies of these phenotypic observations in *Inga* seedling development could be explained by different nutritional regimes or edaphic factors in both forest ecosystems or could be influenced by differences in the level of nutritional outsourcing provided by the establishment of beneficial associations with microorganisms, for instance by the residing fungal communities or specifically through intimate relationships with AMF. These differences as shown in **Figure 2** were not significantly different but are mentioned as an interesting observation which requires further investigation.

In this study, macro and micronutrients concentrations were quantified for the rhizosphere samples collected from the root systems of *Inga* seedlings from terra firme and várzea forest sites to explore the soil nutritional status. As a result of this soil analysis, half of the assessed soil parameters did not present significant statistical differences between várzea and terra firme plots. This could be related to rapidly changing river geography within the Amazon Basin (Wittmann et al., 2006). Thus, areas that are considered drier parts, could become inundated in a short geological timeframe. Furthermore, a higher sample number could be a statistically stronger basis to detect potential differences, as small changes in soil composition are known to have strong impacts on soil microorganism composition, thereby exerting effects not only on AMF populations but even on the plant community structure aboveground. It has been previously recorded that a strong influence of edaphic factors exists on AMF formation and development in acid and nutrient-poor soils in Amazon forest ecosystems (Oliveira and Oliveira, 2010). Therefore, we will discuss both biological trends and significant differences as revealed by the physical and chemical property assessment of terra firme and várzea soils surrounding the roots of *Inga* individuals.

Results of the physical-chemical properties showed that várzea soils, as compared to terra firme soils, presented higher concentrations of all chemical elements except N, Mo, and V, which confirmed that periodic flooding of white-water rivers like the Tiputini river led to increased soil fertility of the várzea floodplains flanking these rivers. Furthermore, the relatively lower N levels in várzea forests could be a result of lixiviation, as nitrogen is a mobile compound and could easily be drained from the forest topsoil layers in várzea forests. Nitrogen is a major nutrient for plant development that frequently limits primary productivity in terrestrial ecosystems (Veresoglou et al., 2012). In highly productive systems which sustain high levels of plant biomass and are limited by nitrogen, such as those observed at the TBS, even small amounts of N gained by trees may give them a competitive advantage. Under these conditions, investing in natural mutualistic associations proves beneficial and could result in AMF recruitment by plants. AMF colonization, temporal and spatial abundance and AMF spore density are positively correlated with soil organic matter and total soluble N (He et al., 2002). AMF have the capacity to acquire N from both inorganic and organic sources, and partially transfer this N to their host plant (Hodge and Storer, 2015). Although overall AMF abundances did not significantly differ between terra firme and várzea soils, there were significant differences in the AMF composition between both forest ecosystems. Whether there is a correlation between the significantly lower N concentrations in várzea, and an AMF composition shift towards species which potentially can exchange higher amounts of N, is difficult to show due to the many unidentified ASVs as shown in **Figure 4**. Furthermore, it would be very interesting to explore whether nitrogen-fixing bacteria show higher abundances in association to the roots of Inga in várzea soils. Cobalt (Co) has a positive effect in promoting both shoot and root biomass, particularly under low P levels. It is possible that high concentrations of this element in várzea forests could have led to a higher shoot to root ratio as observed in **Figure 2**. In Neotropical forests, other elements like Al, Ca, Mg, K, Fe and Mn have been reported to affect colonization and spore numbers of AM fungi (Oliveira and Oliveira, 2010), which is in line with our metabarcoding results as these soil chemical elements (K, Al, Fe, and Cu) explained the highest amount of variation between the composition of the mycobiome and AMF communities of *Inga* in terra firme or várzea forest soils (see PCoA showed in **Figure 6**). Ca is known to be a vital element for the neutralization of soil or water acidity after floodings (Katutis and Rudzianskaite, 2015), but is also linked to both AMF richness and diversity (Guo et al., 2022). The beneficial effects of the AMF for host plants are directly related to the fungus’ ability to supply nutrients by extending the effective root absorption surface areas by incorporating its hyphal network. By doing this, the fungus can explore larger soil volumes and overcome nutrient and water depletion zones near the plant root (Clark and Zeto, 2000). Finally, pH levels in both ecosystems were observed to be acidic (pH range from 3.8 to 5), which could act as a selective force for AMF community structure. Winagraski et al., 2019 reached the conclusion that the important factor shaping the AMF community structure are environmental factors rather host-plant characteristics.

As showed in **Supplementary Table 2**, the richness and homogeneity of each *Inga* rhizosphere sample suggested that there was no difference between either the terra firme and várzea forest ecosystems, nor between the two sampling campaigns. According to Ritter et al. (2020), this is because different areas in the Amazon can present similar levels of species richness but could display different community compositions. There was the exception for homogeneity and richness, measured through the Evenness and Shannon index, respectively, for the sampling period of the ASVs only from the Fungi kingdom, where November 2018 presented greater diversity and richness than in August 2019. The soils of occasionally flooded forests like várzea tend to have a slightly more acidic pH (Randall, 2017). The slight differences in pH levels of terra firme (4.92) and várzea soils (4.65) as encountered in this study, could be responsible for different fungal communities (**Supplementary Table 1**). Analysing the β-diversity of the ITS sequences, both ecosystems clustered differently when analysing the Jaccard, Weighted and Unweighted UniFrac indices. Although fungi are generally less sensitive to changes in pH and have a wide range of pH in which they grow without inhibition of their development, it can be said that the pH and other conditions influence the community structure. Rousk et al. (2010) published that the rhizosphere provides a buffer environment in which changes in the soil environment did not result in variations in the fungal community associated with the rhizosphere. This seemed to be the case in our study, as the results of the beta diversity analysis on Glomeromycotina ASVs showed similar AMF abundances in both ecosystems.

Within the Fungi kingdom, several phyla were identified (**Figure 3**), with the phylum Ascomycota as the most abundant, then followed by Basidiomycota, Rozellomycota, Glomeromycota (now subphylum Glomeromycotina), Zygomycota, and Chytridiomycota, which were present in smaller proportions, in accordance with Zhao et al. (2020). They indicated that Ascomycota portray a faster evolutionary rate and a greater overall diversity of species, which is why they manage to adapt faster to different environments, and they successfully survive in the litter or humus of both terra firme and várzea soils (Richardson et al., 2001). A fungal phylum which was present in higher abundances in the terra firme samples compared to the várzea samples were members of the Blastocladiomycota. They live in muddy soils where they portray a saprotrophic lifestyle, decomposing plant and animal organic matter, or parasitize arthropods (Money, 2016). It is interesting to observe this “aquatic” fungi to be more present in terra firme soils, as based in their lifestyle you might expect them to be more present in seasonally flooded várzea soils. Moreover, members belonging to the Glomeromycotina (former Phylum Glomeromycota) were present in small proportions, and included 13 families, including Glomeraceae, Gigasporaceae, Acaulosporaceae, Diversisporaceae, and 9 previously unidentified families. The Glomeraceae family was the predominant family with a relative abundance of 72.23% of the residing AMF community. Similar trends have been found by Peña-Venegas and Vasco-Palacios (2019), which identified Glomeraceae and Acaulosporaceae as the most common arbuscular mycorrhizal fungi in Amazonian soils due to their associations with species like the members of the genus *Inga*, and as they thrive in warm and humid conditions. As one of the few other mycobiome studies in the Ecuadorian Amazon, which explored the AMF diversity associated to native plants growing in petroleum-polluted sites, Garcés-Ruiz et al., 2019 discovered that Acaulosporaceae dominated the AMF community composition in these oil pits. We show contrasting results for undisturbed sites at the TBS, as we identified members of this family to represent a smaller proportion of the AMF mycobiome. We encourage further studies to confirm whether this composition shift is due to the oil contamination, or for instance the difference in host plants in both studies. The higher presence of species of Glomerceae species which have been recorded to present higher root hyphal densities compared to for example Acaulosporaceae species, could be due to their capacity to provide profound P benefits to their host plants like *Inga* (Deepika and Kothamasi, 2015). Acaulosporaceae are considered stress tolerant and have been reported in acidic soils and under harsh environmental conditions, whereas Gigasporaceae have a slower growth and longer colonization period, once established, they provide nutrients in higher amounts than Glomeraceae or Acaulosporaceae (Goss et al., 2017). Although it requires some expertise to be able to identify AMF spores to the species level, the results of the AMF spore morphological identification using microscopy encountered AMF spores from all three families: Glomeraceae, Acaulosporaceae, and Gigasporacea. This confirmed the outcome of the molecular AMF characterization and strengthened the importance of these groups of mycorrhizae-forming fungi in Neotropical forest soils. One out of four ASVs was annotated to 9 AMF families of unidentified origin, which confirms that the percentage of molecularly identified arbuscular mycorrhizae in the rhizosphere of plants is still very limited and supports the idea to promote to archive more AMF sequences to public databases (Buee et al., 2009; Garcés-Ruiz et al., 2019). Interestingly, results of recent studies in Brazilian forest ecosystems indicate that some arbuscular mycorrhizal fungi are considered omnipresent, whereas others are relatively rare to find (Winagraski et al., 2019). When Fungal ASVs were molecularly analyzed using GNEISS metrics, it was identified that the várzea ecosystem presented a slightly higher Sordariomycetes proportion, while the terra firme ecosystem revealed higher incidences of the *Scedosporium* genus and two taxa of the Nectriaceae family. Furthermore, various taxa of the *Penicillium* genus were present in higher proportions in samples collected in August 2019, compared to samples of November 2018.

In conclusion, the present study describes for the first time the mycobiome associated to the rhizosphere of seedlings of the genus *Inga* in the Tiputini Biodiversity Station in the Ecuadorian Amazon. The rhizosphere of *Inga* seedlings is surrounded by AMF in relatively lower but similar proportions compared to other fungal phyla, but AMF were found in both terra firme and várzea ecosystems and in both sampling seasons. Understanding and evaluating the spatio-temporal dynamics of the root mycobiome, with a specific focus on the role of AMF, could be crucial to understand how undisturbed primary tropical rainforest ecosystems function, and how they influence the underlying mechanisms.

## Data availability statement

The datasets presented in this study can be found in online repositories. The names of the repository/repositories and accession number(s) can be found below: https://www.ebi.ac.uk/ena,PRJEB57077.

## Author contributions

The study was designed by AH-D, PH, and JD. Research permits were managed by SZ and PH. Samples were taken by AH-D, PH, and JD. AH-D and PH prepared the samples for physical chemical soil analysis and amplicon sequencing. VA-G and BP-V conducted the bioinformatics analyses. JD prepared the soil, root, and spore samples and executed the mycorrhizal potential experiments. PH and JD complemented the analyses with their specific expertise and interpreted the overall data. AH-D, PH, JD, VA-G, and BP-V visualized the results and wrote the manuscript. All authors read and approved the final version of the manuscript.

## Supplementary material

Supplementary Figure 1 Temperature and precipitation at the Tiputini Biodiversity Station in the province of Orellana in the Ecuadorian Amazon. Blue columns represent the average monthly precipitation (mm/day) and orange line represent the average monthly temperature (°C) from October 2018 to March 2020. Data collected by Vantage Pro 2 weather station.

Supplementary Figure 2 ITS sequencing of rhizosphere soil samples of juvenile *Inga* plants collected in varzea (V) and terra firme (TF) ecosystems. Taxa bar plot representing ASVs of all eukaryotic microorganisms amplified by ITS.
