# Supplementary material for: Exploring the mycobiome and arbuscular mycorrhizal fungi associated with the rizosphere of the genus Inga in the pristine Ecuadorian Amazon
Source: Front Fungal Biol. 2023 Mar 3;4:1086194. doi: 10.3389/ffunb.2023.1086194 (PMC10512398; doi:10.3389/ffunb.2023.1086194)
Supplement: Supplementary file 1 [file Table_1.docx]

**Supplementary Table 1: Physical-chemical soil properties analysis for várzea and terra firme.** Soil samples presented a normal distribution using a Kolmogórov-Smirnov test (K-S). Critical K-S test value for all samples was 0.41. A Student’s T test was performed to calculate statistical significances between physical-chemical soil properties for both ecosystems, using α=0.5. Abbreviations and symbols: ns = no significance, * = P ≤ 0.05, ** = P ≤ 0.01, *** = P ≤ 0.001.

| **Macronutrients** | **Units** | **Várzea** | **Terra Firme** | **K-S test Norm.** | **T test** | **Significance** |
| --- | --- | --- | --- | --- | --- | --- |
|  |  |  |  |  | **P value** |  |
| Nitrogen (N) | total (%) | 0.4 | 0.6 | 0.15 | 0.024 | * |
| Phosphorus (P) | mg/kg | 678.3 | 518.5 | 0.11 | 0.043 | * |
| Potassium (K) | mg/kg | 7649.3 | 6732.4 | 0.8 | 0.063 | ns |
| Magnesium (Mg) | mg/kg | 4499.8 | 2901 | 0.4 | 0.01 | ** |
| Calcium (Ca) | mg/kg | 3418.7 | 1336.1 | 0.22 | 0.004 | ** |
|  |  |  |  |  |  |  |
| **Micronutrients** | **Units** | **Várzea** | **Terra Firme** | **K-S test Norm.** | **T test** | **Significance** |
|  |  |  |  |  | **P value** |  |
| Copper (Cu) | mg/kg | 31.18 | 23.18 | 0.11 | 0.007 | ** |
| Zinc (Zn) | mg/kg | 120.22 | 108.98 | 0.18 | 0.25 | ns |
| Iron (Fe) | mg/kg | 34710.06 | 28798.75 | 0.16 | 0.073 | ns |
| Manganese (Mn) | mg/kg | 1000.81 | 217.15 | 0.16 | 0.003 | ** |
| Molybdenum (Mo) | mg/kg | 1.45 | 4.06 | 0.14 | 0.0003 | *** |
|  |  |  |  |  |  |  |
| **Others** | **Units** | **Várzea** | **Terra Firme** | **K-S test Norm.** | **T test** | **Significance** |
|  |  |  |  |  | **P value** |  |
| pH | n.a. | 4.65 | 4.92 | 0.16 | 0.174 | ns |
| Na | mg/kg | 4522.95 | 1464.13 | 0.18 | 0.006 | ** |
| Conductivity | µs/cm | 163.12 | 107.74 | 0.28 | 0.313 | ns |
| Aluminum (Al) | mg/kg | 75499.2 | 61283.8 | 0.11 | 0.062 | ns |
| Lead (Pb) | mg/kg | 9.78 | 9.92 | 0.1 | 0.4 | ns |
| Vanadium (V) | mg/kg | 158.29 | 204.05 | 0.09 | 0.026 | * |
| Nickel (Ni) | mg/kg | 26.99 | 25.15 | 0.13 | 0.353 | ns |
| Chromium (Cr) | mg/kg | 77.15 | 74.31 | 0.13 | 0.376 | ns |
| Cobalt (Co) | mg/kg | 22.36 | 10.31 | 0.22 | 6.897 E^-06^ | *** |
| Cadmium (Cd) | mg/kg | 4.04 | 3.42 | 0.11 | 0.08 | ns |
| Barium (Ba) | mg/kg | 496.24 | 293.03 | 0.16 | 0.001 | *** |
