# Supplementary material for: Exploring the mycobiome and arbuscular mycorrhizal fungi associated with the rizosphere of the genus Inga in the pristine Ecuadorian Amazon
Source: Front Fungal Biol. 2023 Mar 3;4:1086194. doi: 10.3389/ffunb.2023.1086194 (PMC10512398; doi:10.3389/ffunb.2023.1086194)
Supplement: Supplementary file 2 [file Table_2.docx]

**Supplementary Table 2. Overview of statistics for alpha and beta diversity corresponding to ITS ASVs.** The Kruskal-Wallis (H) test was applied to calculate alpha diversity, whereas the PERMANOVA (F) test was used to calculate the beta diversity. As variables of interest, both “ecosystem” and “sampling date” were analyzed (*values ​​p<0.05 were statistically significant).

| Diversity type | Alpha diversity | | | | | | Beta diversity | | | | | | | |
| --- | --- | --- | --- | --- | --- | --- | --- | --- | --- | --- | --- | --- | --- | --- |
| Indexes | **Evenness** | | **Faith** | | **Shannon** | | **Bray Curtis** | | **Jaccard** | | **Weighted UNIFRAC** | | **Unweighted UNIFRAC** | |
| Statistical test | **H** | **p value** | **H** | **p value** | **H** | **p value** | **F** | **p value** | **F** | **p value** | **F** | **p value** | **F** | **p value** |
| Total ASVs |  |  |  |  |  |  |  |  |  |  |  |  |  |  |
| Ecosystem type | 0,41 | 0,51 | 0,41 | 0,51 | 0,06 | 0,79 | 1,30 | 0,13 | 1,13 | 0,002* | 2,0 | 0,04* | 1,17 | 0,04* |
| Sampling date | 2,39 | 0,12 | 1,14 | 0,69 | 1,66 | 0,19 | 2,69 | 0,002* | 1,01 | 0,24 | 4,13 | 0,002* | 1,12 | 0,11 |
| Fungal ASVs |  |  |  |  |  |  |  |  |  |  |  |  |  |  |
| Ecosystem type | 0,09 | 0,77 | 0,09 | 0,77 | 0,02 | 0,88 | 1,12 | 0,27 | 1,13 | 0,003* | 1,17 | 0,25 | 1,24 | 0,03* |
| Sampling date | 6,19 | 0,01* | 0,34 | 0,56 | 4,20 | 0,04* | 2,92 | 0,003* | 1,04 | 0,10 | 6,30 | 0,003* | 1,11 | 0,14 |
| *Glomeromycota* ASVs |  |  |  |  |  |  |  |  |  |  |  |  |  |  |
| Ecosystem type | 0,54 | 0,46 | 0,06 | 0,81 | 0,06 | 0,81 | 1,09 | 0,08 | 1,05 | 0,12 | 2,01 | 0,07 | 1,47 | 0,11 |
| Sampling date | 0,06 | 0,81 | 2,94 | 0,09 | 0,96 | 0,33 | 1,07 | 0,17 | 1,03 | 0,23 | 0,77 | 0,62 | 1,22 | 0,23 |
