# Supplementary figures and images for: Exploring the mycobiome and arbuscular mycorrhizal fungi associated with the rizosphere of the genus Inga in the pristine Ecuadorian Amazon
Source: Front Fungal Biol. 2023 Mar 3;4:1086194. doi: 10.3389/ffunb.2023.1086194 (PMC10512398; doi:10.3389/ffunb.2023.1086194)

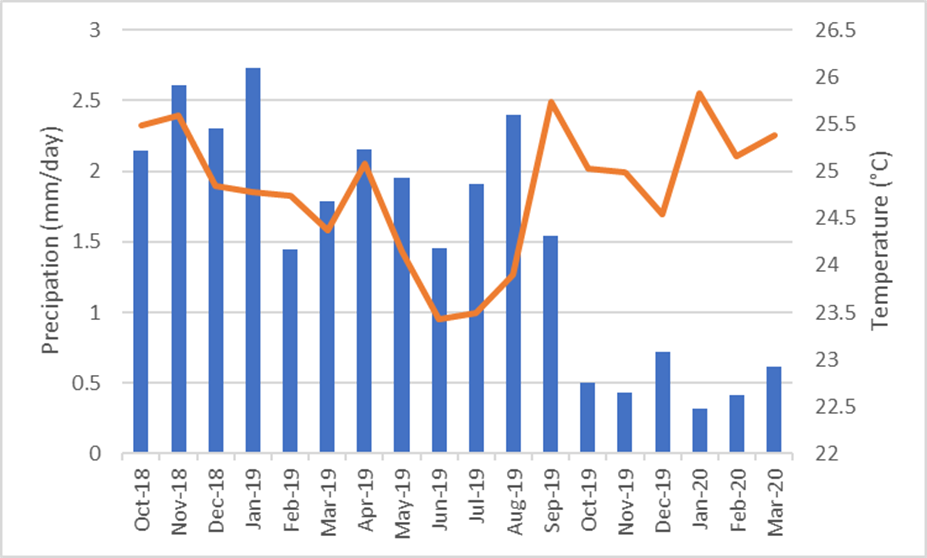

Supplement: Supplementary Figure 1 — Temperature and precipitation at the Tiputini Biodiversity Station in the province of Orellana in the Ecuadorian Amazon. Blue columns represent the average monthly precipitation (mm/day) and orange line represent the average monthly temperature (°C) from October 2018 to March 2020. Data collected by Vantage Pro 2 weather station. [file Image_1.tif]

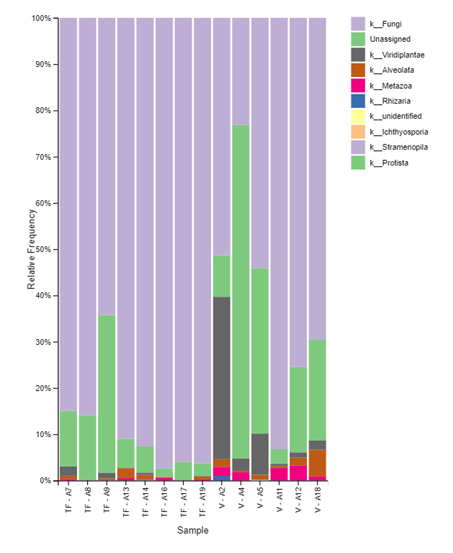

Supplement: Supplementary Figure 2 — ITS sequencing of rhizosphere soil samples of juvenile Inga plants collected in varzea (V) and terra firme (TF) ecosystems. Taxa bar plot representing ASVs of all eukaryotic microorganisms amplified by ITS. [file Image_2.tif]
